# Microalgal cultivation for biofertilization in rice plants using a vertical semi-closed airlift photobioreactor

**DOI:** 10.1371/journal.pone.0203456

**Published:** 2018-09-12

**Authors:** Michael Jochum, Luis P. Moncayo, Young-Ki Jo

**Affiliations:** Department of Plant Pathology & Microbiology, Texas A&M University, College Station, Texas, United States of America; The Education University of Hong Kong, HONG KONG

## Abstract

Nitrogen (N) is one of the most important limiting factors in conventional rice (*Oryza sativa*) production, which heavily relies on synthetic fertilizers. In this study, we researched on the development and use of a vertical semi-closed airlift photobioreactor (PBR) for microalgal cultivation and subsequently determined the efficacy of microalgae-based fertilizers to rice plant growth. The PBR system was developed to produce two strains of N_2_-fixing cyanobacteria (*Anabaena* sp. UTEX 2576, *Nostoc muscorum* UTEX 2209S), and a polyculture of *Chlorella vulgaris* (UTEX 2714) and *Scenedesmus dimorphus* (UTEX 1237). When these biofertilizers were evaluated for rice under the greenhouse conditions, results showed that the rice plant heights treated with polyculture-based microalgal biomass were similar to or better than the urea treatment. The effects of the inoculation of the N_2_-fixing cyanobacterial inoculation on seedling growth was not statistically significant. In conclusion, the vertical semi-closed system PBR cultivation method developed in this study proved to be a simple and effective method for cultivating microalgae. Demonstration of the reliable production system for N_2_-fixing cyanobacteria and chlorophytes at a medium scale could potentially open the future application of microalgal biofertilizers in rice production.

## Introduction

Nitrogen (N) is one of the most important limiting factors in rice (*Oryza sativa* L.) production. Since rice is grown under anaerobic conditions, application efficiency of inorganic N is low due to losses by ammonia volatilization and denitrification[1]. Therefore, without proper N uptake, optimal rice production cannot be certain, even with employing all other management practices and planting high-yielding modern varieties [2,3]. Conventional rice production heavily relies on large applications of synthetic nitrogen fertilizers that adversely impact on environment through contribution to eutrophication zones, and methane emissions from rice fields [4]. One example of sustainable alternatives is the biological application of organic N derived from N_2_-fixing cyanobacteria in tropical [5–8] and temperate rice-growing regions [9,10]. In addition to N fixation, cyanobacteria benefit rice plant health, resulting from nutrient assimilation and release upon cyanobacterial decomposition, increased level of organic carbon in the soil [7,11], and excretion of extracellular compounds like polysaccharides [12] and peptides [13] which facilitate a rapid regeneration and improvement of soil physical properties [7].

Despite previous reports about nutritional improvement to rice and socioeconomic benefits such as nutrient recycling, microalgae-based fertility has not been adopted for commercial rice production in the United States, possibly due to a lack of research on economic and reliable cultivation of microalgae for applications in rice cropping systems. The objectives of this study are to develop a method for autotrophic microalgal cultivation system in a semi-closed PBR and to evaluate the effects of microalgal fertilizer applications in rice plants.

## Materials and methods

### Photobioreactor design

**Fig 1:**
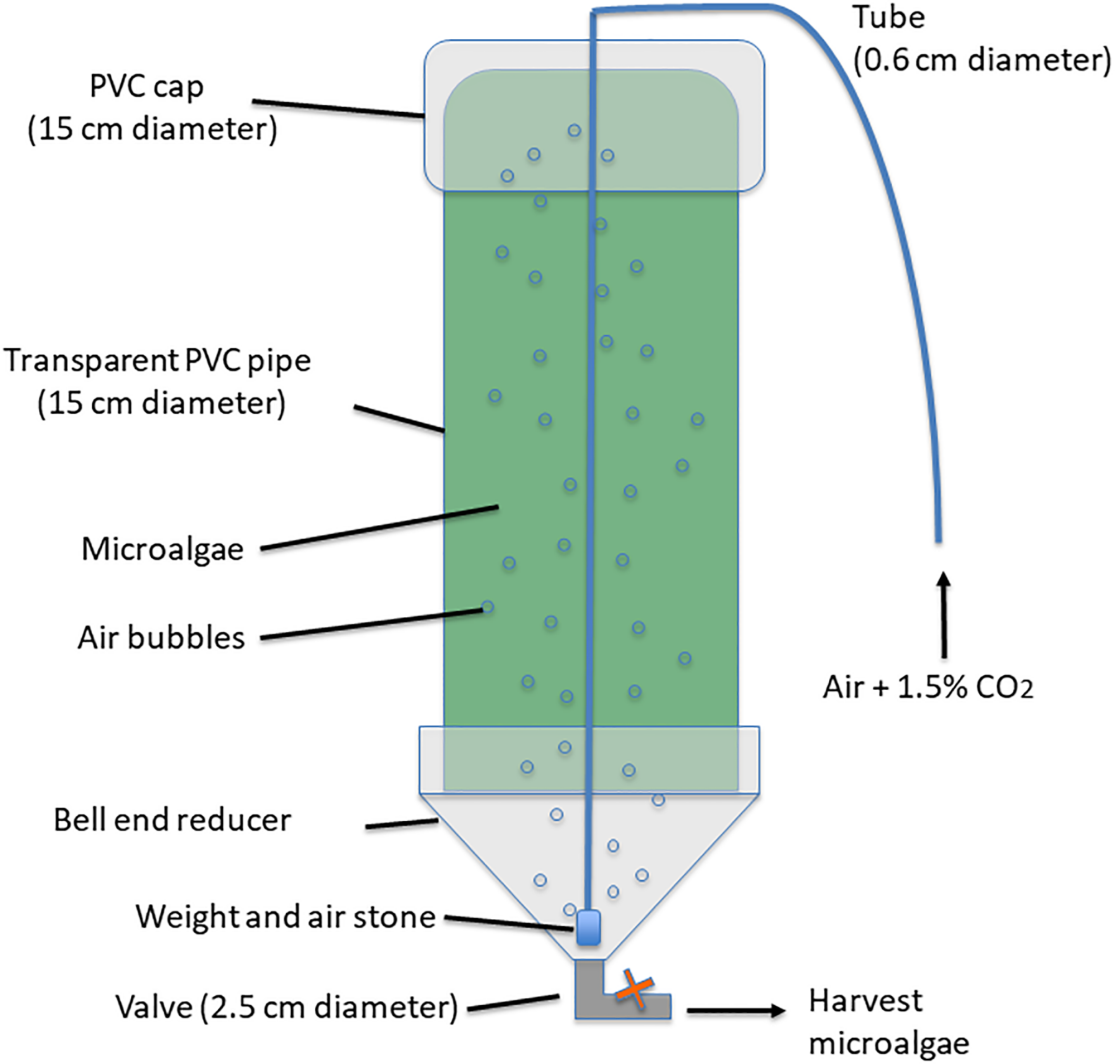
Schematic diagram of the photobioreactor developed in this study.

A semi-closed PBR cultivation apparatus was designed using a 15 cm in diameter clear polyvinyl chloride (PVC) tube placed vertically into a schedule 40 bell end reducer connected to a 2.5-cm ball valve (Fig 1). A 0.65-cm airline coupled to a 250 g weight terminated with a porous (100 μm) brass air stone was introduced from the top lid and lowered into the bottom center of the tube to provide constant aeration at 15 L per minute via air compressor. The compressed air was mixed with 1.5% CO_2_ through a regulator. The total volume of each PBR was 15 L. Each PBR was surface sanitized using a 10% bleach solution followed by three successive washes with water before use.

### Microalgae

N_2_-fixing cyanobacterial cultures of *Anabaena* sp. (UTEX 2576) and *Nostoc muscorum* (UTEX 2209S) were provided by the UTEX Culture Collection of Algae at the University of Texas at Austin. These strains of filamentous cyanobacteria can fix atmospheric N_2_ through specialized heterocyst cells and grow in the nitrogen-deplete medium BG-11-0 (0.23 mM K_2_HPO_4_, 0.3 mM MgSO_4_·7H_2_O, 0.24 mM CaCl_2_·2H_2_O, 0.031 mM citric acid·H_2_O, 0.021 mM ferric ammonium Citrate, 0.0027 mM Na_2_EDTA·2H_2_O, 0.19 mM Na_2_CO_3_, and 1 mM sodium thiosulfate pentahydrate). In addition to the cyanobacteria, the robust chlorophyte strains, *Chlorella vulgaris* (UTEX 2714) and *Scenedesmus dimorphus* (UTEX 1237), were used for PBR growth analysis and production of microalgal biomass to be used as a biofertilizer. Long-term storage cultures of the microalgae were maintained on agar slants containing BG-11-0 for cyanobacteria or MB3N for chorophytes at ambient room temperature with a maximum light intensity of 300 μE m^-2^ s^-1^ from fluorescent lamps with an automated light/dark cycle of 12h/12h.

### Algae cultivation

Each N_2_-fixing cyanobacterial strains (UTEX 2576 and UTEX 2209S) were inoculated from long term storage agar slants and scaled up to 1 L BG-11-0 liquid medium [14] in a Erlenmeyer culture flask while maintained at 25°C with a maximum light intensity of 300 μE m^-2^ s^-1^ from fluorescent lamps with an automated 12-hour light and dark cycle, and constant aeration of normal air infused with 1.5% CO_2_. Once the inoculum reached a sufficient density in the lab based on light spectroscopy, each 1 L cyanobacterial culture was then transferred to one of five PBRs under an ambient light condition (25 ± 5°C) and filled with BG-11-1 liquid medium (BG-11-0 plus 17.6 mM NaNO_3_) as a completely randomized experimental design. Cultures volumes were increased in a batch method up to 7.5 L by the addition of 50% volume with fresh media. Once the volume reached the total of the PBR, the medium was either transferred into an adjacent PBR for repeat cultivation or transitioned to BG-11-0 to induce nitrogen deprivation and heterocyst formation. Cultures were allowed to grow until a sufficiently dense inoculum that was ready to be harvested. Harvested cultures of N_2_-fixing cyanobacteria strains were kept in room temperature and used within 24 hours for the greenhouse evaluation.

For producing microalgal biomass, *C*. *vulgaris* (UTEX 2714) and *S*. *dimorphus* (UTEX 1237) were used. Each strain was retrieved from long term storage agar slants and scaled up to 1 L in an Erlenmeyer flask containing a nitrogen replete medium which mimicked the (MB3N) [15] using agricultural grade synthetic fertilizers as a scalable substitute reagent. Cultures were incubated at 25°C with a maximum light intensity of 300 μE m^-2^ s^-1^ from fluorescent lamps with an automated 12-hour light and dark cycle, and constant aeration of normal air infused with 1.5% CO_2_. Once the inoculum reached a sufficient density in the lab based on light spectroscopy, 1 L of each culture was then transferred together to one of five PBRs under an ambient light condition (25 ± 5°C) and filled with modified MB3N. Cultures volumes were increased in a batch method up to 15 L as a completely randomized experimental design. Once the maximum volume of the PBR was obtained, the medium was either transferred into an adjacent PBR for repeat cultivation or harvested. Harvested microalgal biomass was then concentrated via centrifugation and placed in a -80°C freezer for cell lysis and storage. Organic nutrient concentrations were determined by total Kjeldahl [16] and inductively coupled plasma methods [17].

Microalgal growth was measured via optical density (OD) obtained using a Beckman Coulter DU 800 ultraviolet (UV)-visible spectrophotometer equipped with light scattering plates. One milliliter of culture was place into a 1 cm diameter polystyrene cuvette alongside another cuvette filled with the appropriate sterile medium as a blank. The OD of each dilution series of algae derived from a stock culture was measured at absorbance of 680nm [18]. Algal dry biomass was obtained via vacuum filtration onto pre-tared 47mm Whatman glass fiber filters, followed by washing with an equal volume of 0.5 M ammonium bicarbonate. Filters were then transferred to an aluminum-weighing dish, and dried in an oven at (110°C) overnight, cooled down in a vacuum desiccator, and then reweighed to a constant weight [19] Growth rates were determined by the correlation between dry mass and light spectroscopy OD measurements [20].

### Greenhouse evaluation

Greenhouse evaluations of rice production consisted of three independently conducted experiments. Each experiments was a completely randomized design with 9 to 18 replications (plants) and repeated once. Two experiments (Exp 1 and 2) were conducted in a greenhouse at Texas A&M University, College Station, and one experiment (Exp 3) was conducted in a greenhouse at the University of Texas at Austin. Seeds of the rice cultivar ‘Cocodrie’ were surface sterilized for 5 minutes in 3% sodium hypochlorite solution, and then washed three times with distilled water. The seeds kept in Petri plates filled with distilled water inside an incubator at 28°C. After 3 days, germinated seeds were planted in a 2.5-L pot containing a soil mix of League soil (pH 5.5, 3.2% sand, 32.4% silt, 64.4% clay, and 3.8 to 4.8% organic matter) and potting mix (Sunshine Professional Growing Mix, Sun Gro Horticulture, Agawam, MA), in a ratio of 1:1 (v/v). Subsequent seedling growth was made to become uniform after removing plants different from the growth of the majority. The pots were placed into polycarbonate totes (60 cm length × 30 cm width × 15 cm height), and the fertilizer treatments were applied. Plant heights from the soil line to the tallest leaf of 9 to 18 plants were measured weekly. The greenhouse condition was set to a 25 ± 10°C under natural light conditions.

In Exp 1, rice plants [21 days after planting (DAP)] were treated with 2 L cyanobacterial liquid culture of *Anabaena* sp. [OD at 680 nm wavelength (OD_680_) = 1.14]; 2 L *N*. *muscorum* (OD_680_ = 0.99); 7.6 g microalgal biomass of *C*. *vulgaris and S*. *dimorphus*; 3 g urea (at the rate of 190 kg N ha^-1^) dissolved in water; or water that served as the non-treated control. The treatments were added to totes, and the water volume of each tote was made at 15 L to flood the pots. Water was weekly refilled to maintain at a level above 7.5 L.

In Exp 2, microalgal fertilizer treatments were applied twice to plants at 7 and 28 DAP. At each application, rice seedlings were treated with 2 L cyanobacterial liquid culture of *Anabaena* sp.; 2 L *N*. *muscorum*; 15.2 g microalgal biomass of *C*. *vulgaris and S*. *dimorphus*; or water that served as the non-treated control. In the case of urea treatment, 3 g urea was applied only at the first application. OD_680 nm_ of *Anabaena* sp. was 0.26 and 0.18 for the first and second application, respectively; OD_680 nm_ of *N*. *muscorum* was 0.15 and 0.03 for the first and second application, respectively. Water volume of each tote was maintained to flood the pots as described above.

In Exp 3, the volume of cyanobacteria inoculum was increased to 15 L. Plants (7 DAP) were treated with 15 L *Anabaena* sp. (OD_680_ = 1.14); 15 L *N*. *muscorum* (OD_680_ = 0.30); 15.2g microalgal biomass of *C*. *vulgaris* and *S*. *dimorphus*; 3 g urea; and water as the non-treated control. Water volume of each tote was maintained as aforementioned.

### Statistical analysis

Microalgal growth in PBR and rice plant height data from the greenhouse experiments were analyzed by SAS 9.3 (SAS Institute Inc., Cary, NC). For microalgal growth, linear models were created using PROC REG to regress OD and microalgal dry weight against the incubation time. For evaluation of microalgal fertilization treatments for rice seedlings, three experiments (Exp 1, 2 and 3) were analyzed independently. Each experiment was repeated once, and a two-tailed *F* test for equality of variances was used to determine if the two datasets could be combined. The data were subjected to analysis of variance (ANOVA). Differences between treatment means at given DAP were determined using Fisher’s protected least significance difference (LSD) at *P* = 0.05.

## Results

### Algae cultivation

The PBR successfully cultivated three N_2_-fixing cyanobacterial strains (UTEX 2576 and UTEX 2209S) and chlorophyta based on polyculture strains of *C*. *vulgaris* and *S*. *dimorphus* within a 6-day timeframe. The polyculture *C*. *vulgaris* and *S*. *dimorphus* microalgal biomass was an average dry weight of 38 g L^-1^ and an N-P-K ratio of 4-6-5. Adequate lighting, strong and continuous aeration, and a smooth inner diameter extrusion of the transparent PVC pipe produced very little biofilm formation on the inner surface of the pipe, therein allowing the cultures to grow to a stationary phase before harvest. Batch gravity harvesting of microalgae via opening the bottom valve and transferring the microalgae culture into 19-L carboys proved to be a simple and efficient process. The semi-closed PBR design prevented contamination by algal predators or competitors during the cultivation and minimized evaporation.

**Fig 2:**
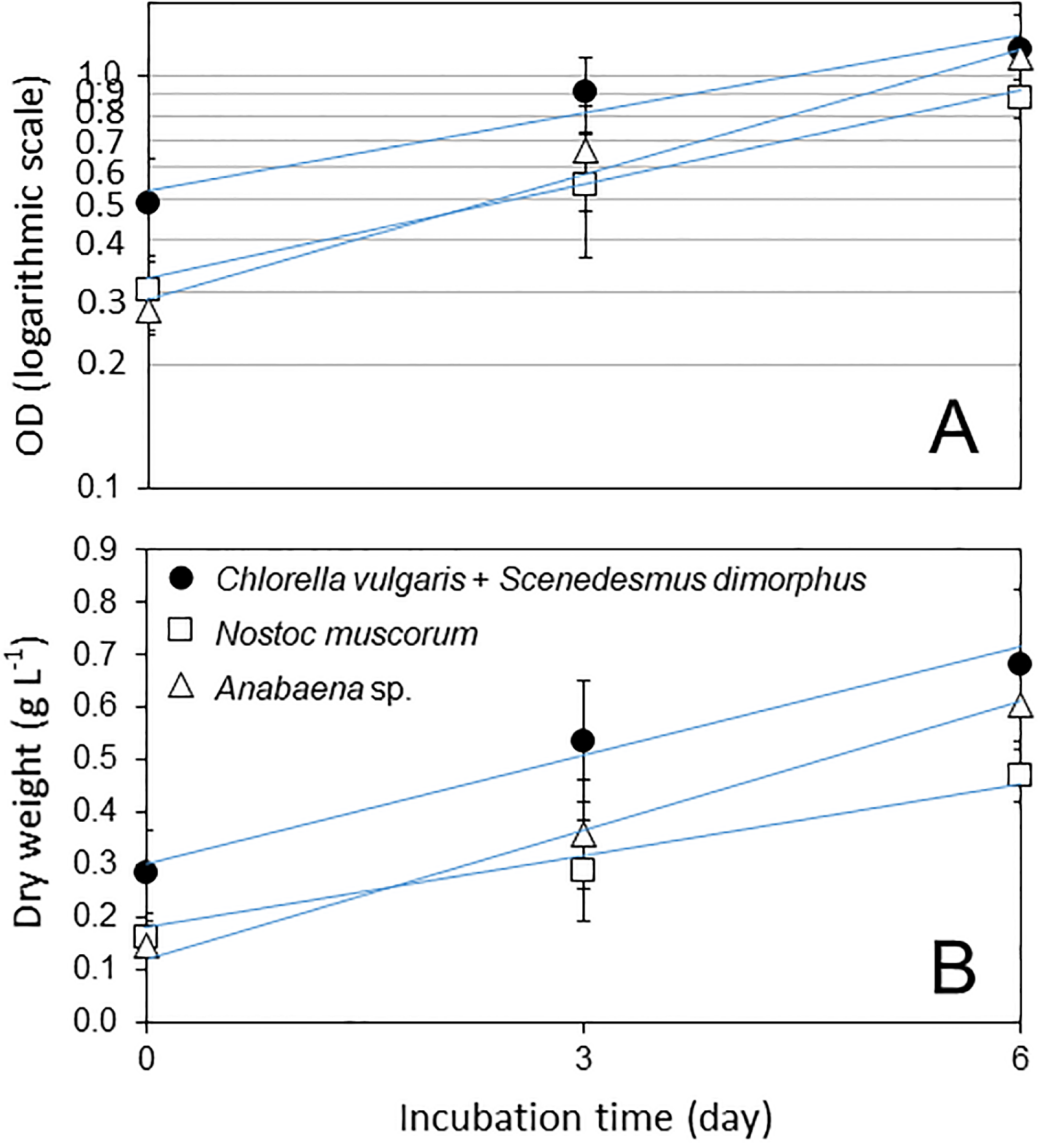
Growth of algae in the 15 L photo bioreactor (PBR). Time-course increase of optical density (OD) at 680 nm wavelength (A) and biomass as dry weight (B) of algae. Mean values from five PBR units with standard deviation are presented.

All linear models for microalgal growth were significant (*P* ≤ 0.0002; S1 and S2 Tables). The growth of N_2_-fixing cyanobacterial strains (UTEX 2576 and UTEX 2209S) and polyculture strains of *C*. *vulgaris* and *S*. *dimorphus* was exponential during the first 6 days as indicated as a linear phase in logarithmic plots in OD_680nm_ (Fig 2A). Algae biomass increased in a linear function of dry weight per day as 51.6% for *Anabaena* sp., 32.0% for *N*. *muscorum*, and 22.9% for the polyculture of *C*. *vulgaris* and *S*. *dimorphus* during the first 6 days (Fig 2B).

### Greenhouse evaluation

**Fig 3:**
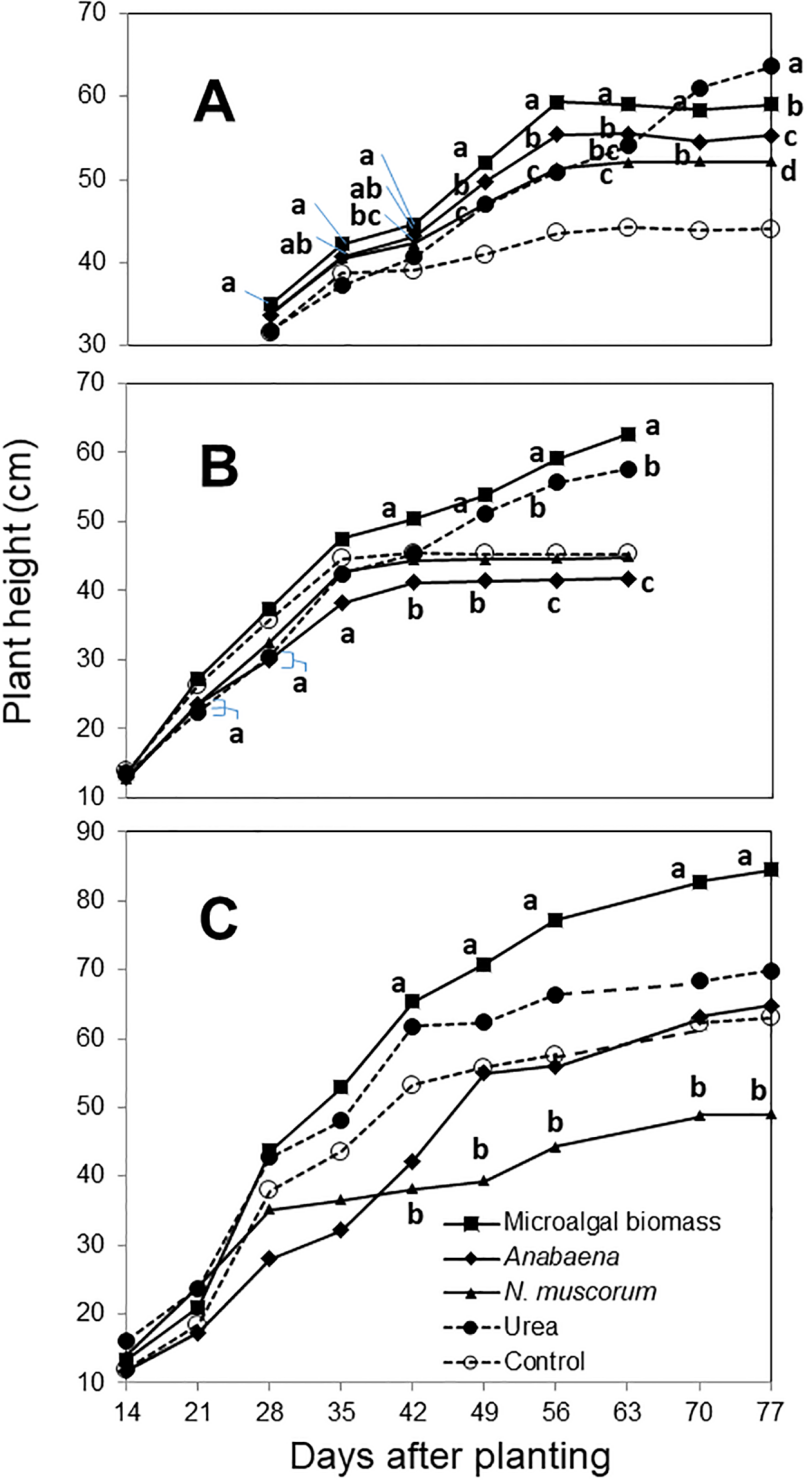
Time-course effect of four different fertilization treatments on the rice plant height under greenhouse conditions. Live inoculum of N_2_-fixing cyanobacterial strains applied was a mixture of *Anabaena* sp. UTEX 2576 and *Nostoc muscorum* UTEX 2209S. Microalgal biomass was a polyculture of *Chlorella vulgaris* and *Scenedesmus dimorphus*. Treatments with the same letter at the same day after planting are not significantly different according to Fisher’s protected leas significant difference (LSD) test at *P* = 0.05. The letters are presented only for the treatments significantly different from the non-treated control. Exp 1 (A), Exp 2 (B) and Exp 3 (C).

Variances of plant height between repeated datasets per each experiment were not different from each other (*P* > 0.05; S3 Table), and subsequently the combined data were used for further analyses. Significant differences (*P* < 0.0001 at Exp 1, 2, and 3; S3 Table) were detected in plant height among the different treatments at different DAP. In Exp 1 (Fig 3A), all treatments showed a progressive increase in plant height and resulted in significant growth improvement compared with the non-treated control after 49 DAP. During the period between 49 and 56 DAP, plants treated with the microalgal biomass or *Anabaena* sp. resulted in the two highest plant heights, followed by the treatments with urea or *N*. *muscorum*. After 56 DAP, plants treated with urea continued growing, while plants treated with the microalgal treatments (*Anabaena* sp., microalgal biomass, and *N*. *muscorum*) almost ceased vertical growth. At 77 DAP, the effect of urea surpassed those of the microalgal treatments.

In Exp 2 (Fig 3B), all treatments showed a progressive increase in plant height at the early growing stage similar to the non-treated control. After 35 DAP, urea and microalgal biomass treatments continuously boosted plant growth and started to show significant improvement of plant height compared with the non-treated control, while the lowest plant height was observed by *Anabaena* sp. inoculation. Plants treated with microalgal biomass resulted in the tallest height, followed by the urea treatment. *N*. *muscorum* treatment was not significantly different from the control.

In Exp 3 (Fig 3C), plants treated with the microalgal biomass resulted in the tallest plant height from 42 DAP. The treatments of *Anabaena* sp. and urea did not significantly increase plant height compared to the control, while *N*. *muscorum* treatment caused the lowest plant height.

## Discussion

The results and observations from this study provide a first case study to evaluate the efficacy of microalgae-based fertilization for rice plants based on a new PBR system. We demonstrated that microalgal biomass resulted in a significant improvement in plant height under greenhouse conditions and addressed the limitations of the PBR system. The main limiting factors associated with a large scale cultivation of microalgae include capital costs of PBR, contamination of the culture with competitors and predators, stochastic weather patterns that influence light availability and growth kinetics, demand for a large amount of water, and energetic and economic costs associated with harvesting and downstream processing [20]. There are innate difficulties in implementing live N_2_-fixing cyanobacteria for the conventional rice production where high yields are derived from high chemical input. Common management practices for convention rice production likely affect effectiveness of microalgae-based biofertilizers. Addition of mineral N by synthetic fertilizers can limit the biological nitrogen fixation potential by cyanobacteria [21,22]. Frequent use of herbicides also can be detrimental to cyanobacteria introduced [21].

We developed a novel vertical semi-close PBR tailored for microalgae biofertilizer production and demonstrated its potential application for rice cropping system. When compared to the other PBR designs for growing microalgae, this apparatus features the following unique characteristics in applicability and prevention of contamination from competitor algae or predators such as rotifer or chytrid. First, this PBR can be reused between batch harvests, therein decreasing overall costs compared to disposable hanging bag PBR designs. Second, the thin wall transparent PVC pipe contains an ultraviolet protectorant during the extrusion process, therein increasing the lifetime of the clear pipe in an outdoor setting compared to non-protected transparent polyethylene, acrylics, or other plastics commonly used in PBR design. Third, the inner wall of the transparent PVC pipe is smoothed during the extrusion process, therein helping mitigate fouling due to biofilm formation and reducing the times needed during maintenance and cleaning, when compared to traditionally extruded plastics. Forth, when compared to glass PBR, this PBR is more resistance to shattering during installation, cultivation, and maintenance procedures. The thin wall transparent PVC used in this study is resistant to shattering from environmentally damaging effects like hail, which can destroy glass or bagged PBR. Fifth, this PBR is designed with a 7.62-cm radius, which provides an adequate balance between light availability, temperature control, capex, and surface area to volume ratio [20,23,24]. Larger diameter PBR designs can limit culture density due to an increased optical path. In the other extreme, smaller diameter PBR designs can lack enough volume to maintain a homeostatic temperature under full sunlight, therefore causing catastrophic detriment to the culture, as seen in small volume tubular and flat-plate reactor designs. Sixth, the top manifold aeration used in this PBR prevents leaking due to degradation of inlet and accidental siphoning into an air pump due to a power outage or loss of pressure from the aeration pump. The airline can be easily disconnected from the pump and dual-used as a sampling port. By connecting a luer lock syringe to the airline, culture samples can be collected without having to open or expose the culture to the ambient environment, therein helping mitigate contamination events. Seventh, harvesting from the bottom of the bell end reducer allows for a quick and easy process that does not expose the interior of the reactor to the outside environment, which helps mitigate contamination events. Eighth, this PBR uses the modular design to mitigate contamination associated with microalgal production. Because the PBR do not share a common manifold, algal culture is never shared between PBR units in an array. Therefore, if a contamination event occurs in a single reactor, it can be sanitized immediately upon recognition of the contamination, therein protecting the remainig cultivation facility. This modular design is not present in open ponds, serpentine reactors, or other designs that share some form of pumping or harvesting manifolds.

An individual PBR developed in this study could produce 15 L microalgae culture within one week. Production of a polyculture of *C*. *vularis* and *S*. *dimorphus* inside the PBR was effective due to the robust fast growing nature of these strains that do not cause coagulation during culture compared to the filamentous cyanobacteria. Microalgae production in this PBR was consistent and reliable. Peak productivities can be maintainable or achievable under controlled environments without the problems that arise from outdoor open pond algal production systems such as susceptibility to predation, competition from aggressive endemic species, overflowing from rainfall and water loss by evaporation [25]. Another advantage of this PBR was to maintain optimal conditions of microalgae growth by continuous harvest and monitoring. This PBR is designed to harvest in a batch-wise manner prior to stationary phase, defined as the state at which net growth ceases altogether. In this manner, the optimal batch harvest system is designed to maintain the balance of growth of culture during its exponential rate, identified as the inflection point during the exponential growth phase. The culture is harvested prior to reaching its maximum density, the point at which the culture asymptotically begins to reach stationary phase and zero net growth. By harvesting in this manner, a culture density reached that is sufficient for downstream processing and cross-inoculation into another PBR.

One of the biggest challenges associated with the cultivation of microalgae at this scale was obtaining accurate measurements under rapidly changing culturing conditions. Real-time monitoring and enhanced equilibration of the PBR conditions will increase growth rate and improve monetary and energy efficiency to the system. Further improvements in this PBR design include an inline pH meter and a near-infrared (NIR) spectrophotometer for measuring cultivation conditions inside the PBR. Based on real-time information, the important growth parameters, such as CO_2_ input and degree of aeration throughout the tube, can be timely adjusted, therein guiding proper decision-making for addition of fresh nutrients and harvesting of cells. Also, rather than relying on stock media recipes such as BG-11 and BG-11-0 for N_2_-fixing cyanobacteria, optimization of the individual macro-, minor-, and micro-nutrients for a particular microalgal strain can facilitate the cultivation efficiency.

This cultivation technique based on this PBR made it feasible to introduce microalgae-driven fertilizers to promote rice production. We evaluated the two microalgal fertility approaches: microalgae biomass and inoculation of live N_2_-fixing cyanobacteria. The application of microalgae biomass demonstrated its benefit in rice seedling growth. Its effect increased as the amount of applied microalgal biomass increased. This indicates that the use of microalgal biomass can provide a biological option for rice fertility program, which is especially important for organic rice production where synthetic fertilizers cannot be used. However, the effect of the inoculation of live N_2_-fixing cyanobacteria into flooded conditions, where rice plants are adopted, could not be confirmed in this study, addressing the complex ecological and environmental challenges that we have to overcome for future use of cyanobacterial introduction to rice production. Most of all, a better understanding about environmental factors associated with microalgae in rice paddy is necessary to solve the problems in the application of the algal fertilizer.

There have been numerous field studies reporting beneficial effects of N_2_-fixing microalgae inoculation as biofertilizers in rice growing regions in Asia since 1960’s [26]. Among recent examples, cyanobacteria inoculation increased rice yields by 5 to 24.1% in Nepal [27,28] and by 12.3 to 19.5% in India [29]. Cyanobacterial contribution to nitrogen fixation was estimated between 20 to 500 μmol C_2_H_2_ m^-2^ h^-1^ acetylene reducing activity per crop cycle [20,21]. When assuming a 4:1 ratio of acetylene reduction to atmospheric N_2_ fixed, these results can be extrapolated to 0.2 to 50 kg N ha^-1^. Field introduction with *Nostoc* sp. could incorporate 20 kg N ha^-1^ into rice [6]. However, there is still limited information on the dynamics of the transfer of atmospherically fixed nitrogen from cyanobacteria to rice under complexed field conditions [21].

Ineffectiveness of N_2_-fixing cyanobacteria inoculation indicates that the success of N_2_-fixing cyanobacteria introduction is contingent on various biological and environmental factors that limit its benefits [30]. Atmospheric N_2_ fixation by cyanobacteria is a metabolic process that costs high energy consumption and tends to decrease in the presence of exogenous N sources [26]. Subsequent decomposition of bacterial cells into plant available forms of N has to be followed. This process can take time. Environmental conditions associated with biological processes in the decomposition of algae biomass and the release of nutrients may be suboptimal, and so necessary nutrients cannot be provided properly and timely in coordination with plant growth. Meanwhile, abiotic stress, predators, competitors and diseases can reduce cyanobacteria density, therein making initial establishment of inoculated strains and propagation difficult [30]. Rice canopy also affects the growth of cyanobacteria, restricting the penetration of light to the water surface which limit microalgal growth and N fixation of cyanobacteria [31]. In some cases, even an adverse effect of microalgae on plant growth may occur due to the excretion of toxic secondary byproducts or unknown inhibitory factors [32,33].

Conventional rice production may not solely rely on microalgal biofertilizers but its use can add an option of socioeconomical- and ecological-sound alternative in the current rice management system in the United States, providing additional benefits to promote a sustainable rice farming. By utilizing wastewater effluent as a base medium for cultivating microalgae, it is possible to capture nutrients from a point pollution source like municipal water treatment facilities and recycle them into agriculture [34,35]. Performance of N_2_-fixing cyanobacteria inoculation is likely influenced by microalgal strains, the application rate, time and frequency. It will be important to discover indigenous cyanobacterial isolates that are more adapted to the regional environment where they were collected [36], and so their augmentation can be more successful in achieving yield increased as documented in row crops [37,38]. Consecutive inoculation in multiple years may be needed to increase the establishment and propagation of cyanobacteria inoculum in rice fields [26]. The microalgae biofertilizer treatments can also be possibly used in combination with synthetic N fertilizers [26,31,39–42]. Additional benefit of rice cultivation with cyanobacteria is to ameliorate metal toxicity in both soil and rice plants because cyanobacteria play a role in sequestering the toxic metal loads alongside the ecosystem service of N_2_ fixation [21,43]. Thus, cyanobacteria may enhance the edibility and safety of rice cultivated under soil conditions with nitrogen deficiency and toxic metal abundance.

## Conclusions

The vertical semi-closed PBR developed in this study was proved to be an effective method of microalgae cultivation. Such a scale-up cultivation of microalgae makes future increased applications of microalgal fertilizers to the rice production. Growth improvement of rice seedlings by addition of microalgal biomass was observed under the controlled environment, but beneficial effects of N_2_-fixing cyanobacteria inoculation treatment were limited. A potential of microalgae biofertilizer has been suggested, but their implementation in the conventional rice production in the United States still faces a challenge. Further ecological research is needed for better adopting and integrating algae-based fertilizers with current rice fertility programs required for the conventional or organic rice production. The combined use of microalgal biofertilizers and synthetic fertilizers has a merit to achieve sustainable productivity and applicability for rice production.

## Supporting information

S1 Table Linear regression models for microalgal growth by optical density (OD). (DOCX)

S2 Table Linear regression models for microalgal growth by dry weight. (DOCX)

S3 Table Analysis of variance of rice seedling growth after the microalgal treatments. (DOCX)
