# Supplementary material for: Microalgal cultivation for biofertilization in rice plants using a vertical semi-closed airlift photobioreactor
Source: PLoS One. 2018 Sep 12;13(9):e0203456. doi: 10.1371/journal.pone.0203456 (PMC6135494; doi:10.1371/journal.pone.0203456)
Supplement: S1 Table — (DOCX) [file pone.0203456.s001.docx]

**S1 Table. Linear regression models for microalgal growth by optical density (OD).**

| **Microalgae** | **Slope** | **Intercept** | ***R^2^*** | ***P*** |
| --- | --- | --- | --- | --- |
| *Chlorella vulgaris* + *Scenedesmus dimorphus* | 0.146 | -0.6788 | 0.7035 | < 0.0001 |
| *Nostoc muscorum* | 0.181 | -1.2141 | 0.7028 | 0.0002 |
| *Anebeana* sp. | 0.235 | -1.2626 | 0.8910 | < 0.0001 |
