# Supplementary material for: Microalgal cultivation for biofertilization in rice plants using a vertical semi-closed airlift photobioreactor
Source: PLoS One. 2018 Sep 12;13(9):e0203456. doi: 10.1371/journal.pone.0203456 (PMC6135494; doi:10.1371/journal.pone.0203456)
Supplement: S2 Table — (DOCX) [file pone.0203456.s002.docx]

**S2 Table. Linear regression models for microalgal growth by dry weight.**

| **Microalgae** | **Slope** | **Intercept** | ***R^2^*** | ***P*** |
| --- | --- | --- | --- | --- |
| *Chlorella vulgaris* + *Scenedesmus dimorphus* | 0.066 | 0.3043 | 0.6935 | 0.0001 |
| *Nostoc muscorum* | 0.512 | 0.1520 | 0.8001 | < 0.0001 |
| *Anebeana* sp. | 0.076 | 0.1418 | 0.8881 | < 0.0001 |
