# Supplementary material for: Microalgal cultivation for biofertilization in rice plants using a vertical semi-closed airlift photobioreactor
Source: PLoS One. 2018 Sep 12;13(9):e0203456. doi: 10.1371/journal.pone.0203456 (PMC6135494; doi:10.1371/journal.pone.0203456)
Supplement: S3 Table — (DOCX) [file pone.0203456.s003.docx]

**S3 Table. Analysis of variance of rice seedling growth after the microalgal treatments.**

|  | **Source** | **df** | **Mean square** | ***F*** | ***P*** |
| --- | --- | --- | --- | --- | --- |
| Exp 1 | Experiment | 1 | 0.10 | 0.01 | 0.9237 |
|  | Treatment | 4 | 1461.73 | 131.65 | < 0.0001 |
|  | Days after planting (DAP) | 7 | 3763.26 | 338.94 | < 0.0001 |
|  | Treatment x DAP | 28 | 118.01 | 10.63 | < 0.0001 |
|  |  |  |  |  |  |
| Exp 2 | Experiment | 1 | 0.25 | 0.02 | 0.9017 |
|  | Treatment | 4 | 1411.66 | 86.01 | < 0.0001 |
|  | DAP | 7 | 10751.91 | 655.07 | < 0.0001 |
|  | Treatment x DAP | 28 | 149.45 | 9.11 | < 0.0001 |
|  |  |  |  |  |  |
| Exp 3 | Experiment | 1 | 8.09 | 0.05 | 0.8256 |
|  | Treatment | 4 | 8067.08 | 48.49 | < 0.0001 |
|  | DAP | 8 | 25348.78 | 152.38 | < 0.0001 |
|  | Treatment x DAP | 32 | 502.37 | 3.02 | < 0.0001 |
